# Re-shaping pruning improves the dynamic response of centuries-old olive trees to branch-shaker vibrations application

**DOI:** 10.3389/fpls.2023.1155120

**Published:** 2023-04-21

**Authors:** Salvatore Camposeo, Francesco Vicino, Gaetano Alessandro Vivaldi, Simone Pascuzzi

**Affiliations:** Department of Soil, Plant and Food Science, Università di Bari Aldo Moro, Bari, Italy

**Keywords:** accelerometer, impulse excitation, vibratory excitation, spectral analysis, vibrations amplitude

## Abstract

**Introduction:**

The Mediterranean basin is home to centuries-old large olive trees; high-vigor cultivars are widespread, with training forms poorly adapted to mechanical harvesting by trunk/branch shakers. The significant quantity of leaves, the considerable tree height, and the presence of multiple dichotomous hanging branches reduce the transmission of vibrations applied by the branch-shaker machines. Thus, re-shaping pruning may improve the performance of this modern mechanical harvesting method by focusing on removing both the hanging branches and those forming dichotomies. The goal of this study was to evaluate the dynamic responses of large-sized olive trees to pruning (or not) through various field tests under different excitation forces. We hypothesized that more rational pruning could significantly increase vibration transmissions.

**Methods:**

To assess the transmission of vibrations, tests were conducted before and after the pruning on representative trees. Tri-axial accelerometers packed in a small titanium housing were used. Trees were assessed before and after the re-shaping pruning. This study reports the first data about the dynamic behavior of centuries-old tree skeletons, in the context of very large-sized olive trees, while taking into account the effects of two different vibrations application modes: a realistic one represented by the system vibration head-tree, originated when the gripper of a shaking machine wrapped and fastened the main branch of the olive trees, and a more speculative one, represented by a single impulse of a short-duration force originated by a hammer.

**Results:**

After pruning, spectral density increased 10 fold in the tertiary branches of pruned trees (ranging 1.0–10 m s^−2^) compared to that of not-pruned ones (ranging 0.1–1.0 m s^−2^) at frequency >50 Hz under vibration excitation. Moreover, vibrational decay times (120–150 ms) and amplitude (>10^−1^ m s^−2^) were higher under single-impulse excitation.

**Discussion:**

A more rational pruning applied to ancient large-sized olive trees significantly increased the vibration transmission under both impulse and vibratory excitation forces, without affected their typical “look”. Moreover, these insights are helpful in turn in achieving maximum fruit-removal efficiency. These insights could be applied to various horticultural conditions which would improve the economic sustainability of monumental olive trees, a key portion of the Mediterranean landscape and cultural heritage.

## 1 Introduction

Ancient olive trees are indigenous to the Mediterranean basin and characterize the landscape. In the southern Mediterranean, ancient trees comprise 54% of the total olive-growing area, including Tunisia which has over 65-million century-old trees (Calabrese et al., 2012). Ancient olive trees and ancient olive orchards are also present in the EU Mediterranean countries; however, at present, no detailed data are available regarding the surface devoted to this olive-grove typology. This is because the traditional ancient orchards exist in a territorial matrix where even modern olive cropping systems are present and surface estimation is difficult. The presence of ancient olive orchards is reported in different areas of Spain, Portugal, and Greece^1^ (IOC, 2022). In Italy, ancient olive trees are present all over the peninsula, but especially in Apulia (estimated 6 million trees) where they characterize the landscape and are considered crucial for oil production (Calabrese et al., 2012). In Apulia in 2007, a Regional Law was passed to prevent uprooting and to define ancient olive orchards based on the percentage of monumental olive trees present in the field (60% minimum). Therefore, these trees and orchards, which are managed according to traditional agricultural practices, assume greater importance for landscape and cultural heritage. Many of these trees are significantly large in size and thus, are difficult to mechanize; consequently, these olive orchards have low profitability (Pellegrini et al., 2017). Despite its downfalls, harvest mechanization is the key technique for reducing production costs (Tombesi, 1990; Guerrieri et al., 2019). Unfortunately, these ancient tree orchards are not properly managed and therefore show low or no yield (Calabrese et al., 2012), alternate bearing (Mazzeo et al., 2014), and high environmental impact (Pellegrini et al., 2016; Bernardi et al., 2021; Camposeo et al., 2022). In Apulia, ancient olive trees are common in traditional olive growing areas with autochthonous cultivars. These traditional groves are characterized by irregular spacing and low planting density (<90 trees ha^−1^). Severe pruning is executed every 5–6 years or more and only suckers are generally pruned annually (Godini, 2002). The harvest is executed by collecting the olives from the ground every 2 weeks for 4–5 months, for the production of lamp oil (Famiani et al., 2014). The use of shaking machines to mechanize olive harvesting from the crown is particularly significant owing to the exceedingly positive effects in oil-quality improvement (Clodoveo et al., 2014; Sola-Guirado et al., 2018). When the olive tree size is too large, it is better to apply the vibrations to the easily accessible primary branches (Tombesi, 1990). The mechanical harvesting could be conducted by means of self-propelled shaking machines that clamp a main branch at any one time. However, these branch-shaker machines require well-trained and pruned trees. Indeed, significant foliage, considerable branch lengths and multiple and/or hanging branches lead to reduction of the vibration transmissions with a negative impact on the effectiveness for the drupes detachment (Sola-Guirado et al., 2014).

The kinetic energy transferred by the shaker to the main branch of significantly large trees is essentially dissipated because of the complex transmission vibration mechanism among the branches, the leaf drags, and the internal energy losses within the wood and root/soil system (Leone et al., 2015; Ma et al., 2022). Notably, the aerodynamic drag forces of the foliage in the air (hydraulic damping), the interaction of the side branches attached to the main limb (mass damping), and the damping effects within the stem and root system (viscoelastic damping) strongly attenuate the vibration produced by the shaker (Upadhyaya et al., 1981; D’Agostino et al., 2008). To improve the performance of the shakers, several growers executed a re-shaping pruning aimed at removing the branches that are not directly linked to those of the second order. Indeed, third-order branches forming dichotomies are usually eliminated, and the number of second-order branches is reduced. Furthermore, most hanging branches were removed because they do not allow suitable vibration transmissions (Tombesi, 1990; Farinelli et al., 2012). It is known that shaking is a customary technique for mechanical harvesting of tree fruit. Research and development in the past few decades have successfully produced shaking machines capable of harvesting different fruit tree species (Erdogan et al., 2003; Xing et al., 2020). All these studies have been aimed at evaluating the optimal combination of shaking frequency, amplitude, and duration for harvesting specific types of fruit to achieve maximum fruit-removal efficiency (Castro-García et al., 2012; He et al., 2019).

The fundamental concept of shaking harvesting is to transfer a suitable quantity of kinetic energy to fruiting branches to produce a detaching force on the fruit-stem interface which then removes the fruit from the tree (Pezzi and Caprara, 2009; Zhou et al., 2012). During the shaking, each tree reacts differentially to distinct excitation frequencies and amplitudes, and fruit removal takes place when the produced disconnection force exceeds the pedicel-fruit tensile strength (Caprara and Pezzi, 2011; Ehsani et al., 2009). Modern shakers transfer anisotropic multi-directional stress in the geometric form of irregular ellipses; therefore, being non-isotropic, forced stress has preferential directions of action (Du et al., 2012; Torregrosa et al., 2006). The dynamic response of the trees is then broken and is affected by the damping forces that take place along the branches, and by the geometric shape and the arrangement of the branches along the line of action of the gripper (Leone et al., 2015). Furthermore, the shape or morphology of the tree and the distribution of oscillating branch masses significantly influences its dynamic behavior in terms of natural frequencies and damping (Torregrosa et al., 2009; James et al., 2014). Aerodynamic and viscous dampings within the material are the main damping sources inside the trees if the friction among different branches and dissipative mechanisms in the roots–soil system is set aside (Tsatsarelis, 1987; Inman, 1994). As shown, data on the dynamic response to vibrations are available for several fruit tree species and normal-size olive trees.

The goal of this study was to investigate the dynamic responses of ancient large-sized olive trees before and after a re-shaping pruning, under different excitation forces (impulse mode using a hammer and vibratory mode using a branch-shaker machine). We hypothesize that a more rational pruning could significantly increase the vibrations transmission. If yes, the insights could be helpful in turn in achieving maximum fruit-removal efficiency. This is the first contribution to the understanding of the dynamic behavior of centuries-old tree skeletons in the context of the very large-sized olive trees.

## 2 Materials and methods

### 2.1 Orchard characteristics and pruning applied

**Figure 1:**
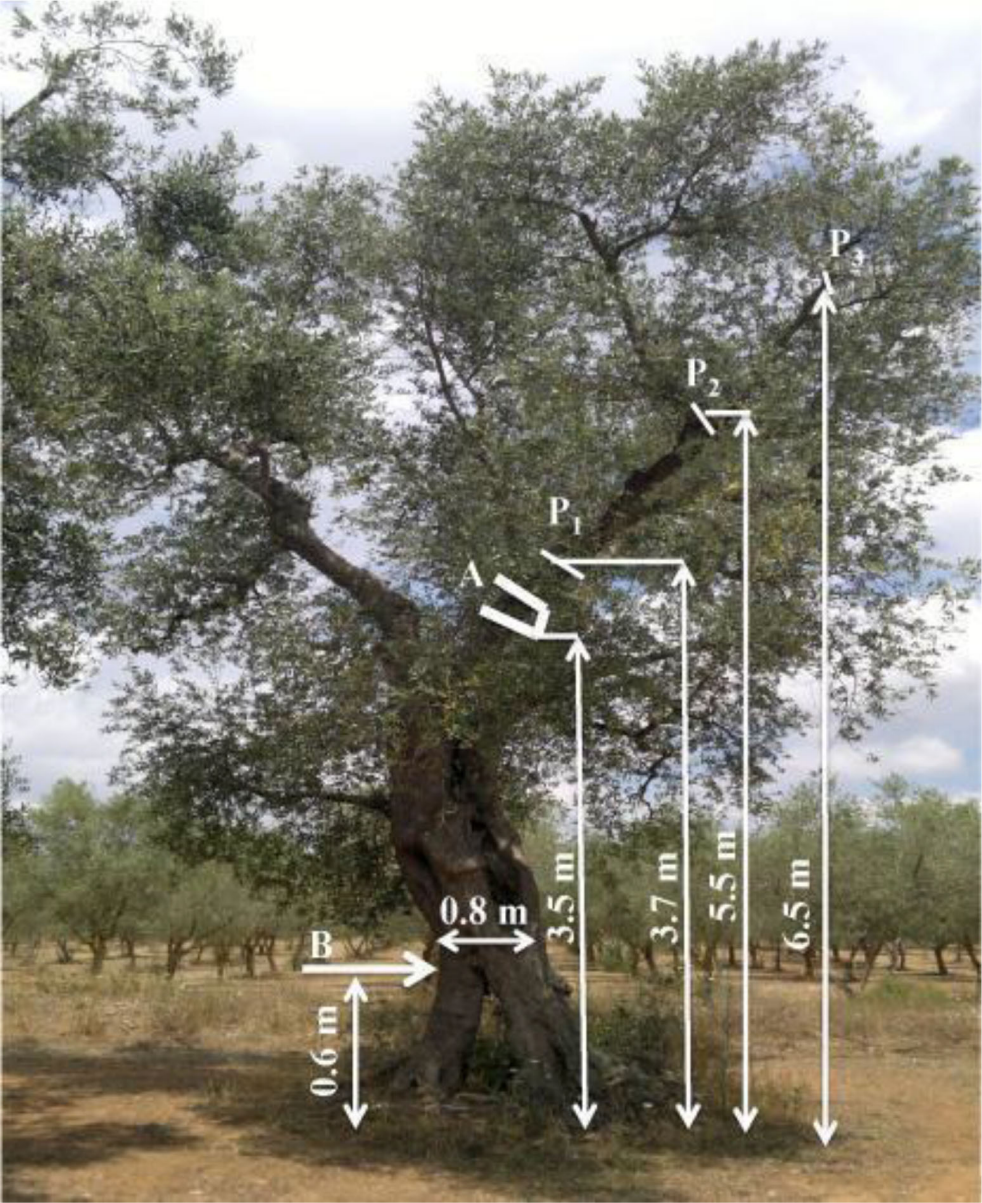
Representative pruned olive tree with its main sizes. P_1_, P_2_, P_3_ are the locations of the accelerometers. **(A)** Position of the gripper of the branch-shaker machine for vibratory excitation. **(B)** Position of the hitting with the hammer for impulse excitation.

The tests were performed on a rainfed olive orchard located near the *Piana degli Ulivi Secolari* area (Apulia, southern Italy; 40° 26’ 35’’ N; 18° 3’ 15’’ E; 37 m a.s.l.) characterized by large-sized olive trees (cv. Cellina di Nardò), spaced 12 m × 12 m apart (70 trees ha^−1^; 142 m^2^ tree^−1^), with an approximate tree age of 250 years. The chosen orchard is considered representative of the Apulian traditional olive training systems (Godini, 2002), of which ten homogeneous vase-shaped trees were randomly selected. The trunk diameter at 1.5 m from the ground was 1.2 m and the height at which the 2 scaffold branches started was 2.8 m. A re-shaping pruning was applied in February 2017 by thinning the tertiary branches including hanging branches and those forming dichotomies. The resulting crown was much less dense with more upright branches and without dichotomies (**Figure 1**).

### 2.2 Biometry

Tree height and width were measured on all the ten selected trees just before and after the re-shaping pruning. Total fresh weight (kg tree^−1^) of the biomass removed by the pruning from each tree was determined, separated, and measured depending on diameter (less and over than 5 cm).

### 2.3 Measure chain

To assess the transmission of vibrations, tests were conducted before and after the pruning on 3 representative trees, those showing very similar skeleton among the ten selected. Tri-axial accelerometers AP 80 (Technology International, Netherlands) packed in a small titanium housing were used. The main technical characteristics included amplitude range 2500 g root mean square (RMS); mechanical shock limit 5000 g peak; frequency range (+/−1 dB) 0.5–2000 Hz; and resonant frequency >55 kHz. The measure chain employed to conduct the experimental tests involved the following components: i) low-noise signal conditioners Mesa C14 (Mesa, USA); ii) channel analyzers 01DB (ACOE, France); iii) personal computer equipped with software dBFA (01dB, Italy). Each acquisition channel was calibrated with an accelerometer calibrator (RION VE-10) before beginning measurements. The selected trees were subjected to both impulse- and forced-vibratory excitations to evaluate the transmission inside the branches of damped-free and forced vibrations, respectively, before and after pruning.

### 2.4 Impulse excitations application

**Figure 2:**
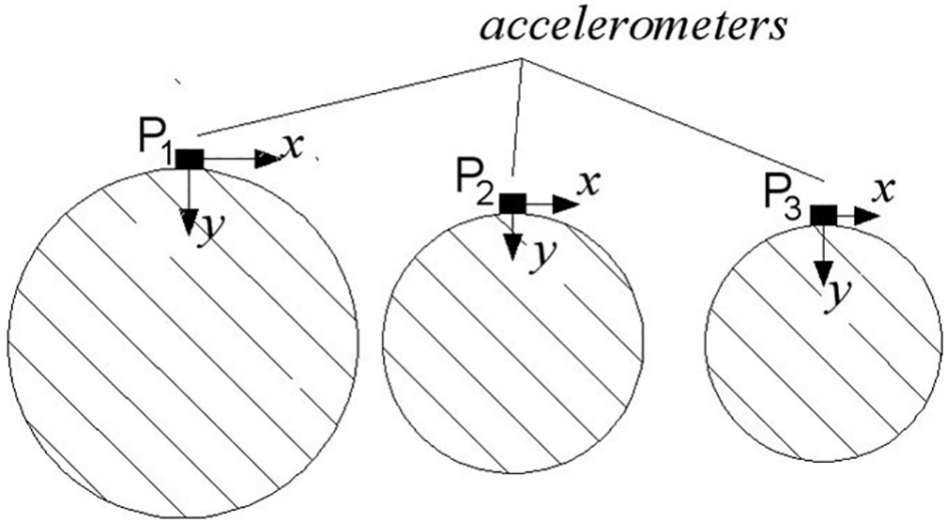
Arrangement of the accelerometers (P_1_, P_2_, and P_3_) in the cross section of the branches, with *x* axis always parallel to the soil.

The impulse excitations were generated by hitting the selected trees with a steel stick used as a hammer at about 60 cm from the ground (B position in **Figure 1**). Shots were given by the same operator. The acceleration values (m s^−2^) after the tree hitting were measured through 3 accelerometers, AP 80 (herein after referred to as P_1_, P_2_, and P_3_); the corresponding frequency responses have been analyzed. The accelerometers were fixed at 3 different points on the same quadrant of tree to obtain a simultaneous measurement of the whole-branch acceleration response to input excitations. One accelerometer was located on each branch order (P_1_, P_2_, and P_3_). After pruning, the procedure was repeated (**Figure 1**). The accelerometers were arranged so that data could be obtained for 2 axes (*x* and *y*) that were mutually orthogonal in the cross section of the branches with *x* axis always parallel to the soil (**Figure 2**). Furthermore, only 2 of the 3 available channels were considered for each accelerometer, neglecting the data pertinent to the perpendicular axis (*z*). This option allows us to analyze only the vibrations in the plane parallel to the ground, which was taken as the reference one (Leone et al., 2015).

The data were then processed by the dBFA software which allowed us to obtain the amplitude RMS (m s^−2^) of the signal, frequency range (Hz), and the decay time (ms) of the impulse excitation in the points of the branches where the accelerometers were placed.

### 2.5 Forced vibratory excitations application

**Figure 3:**
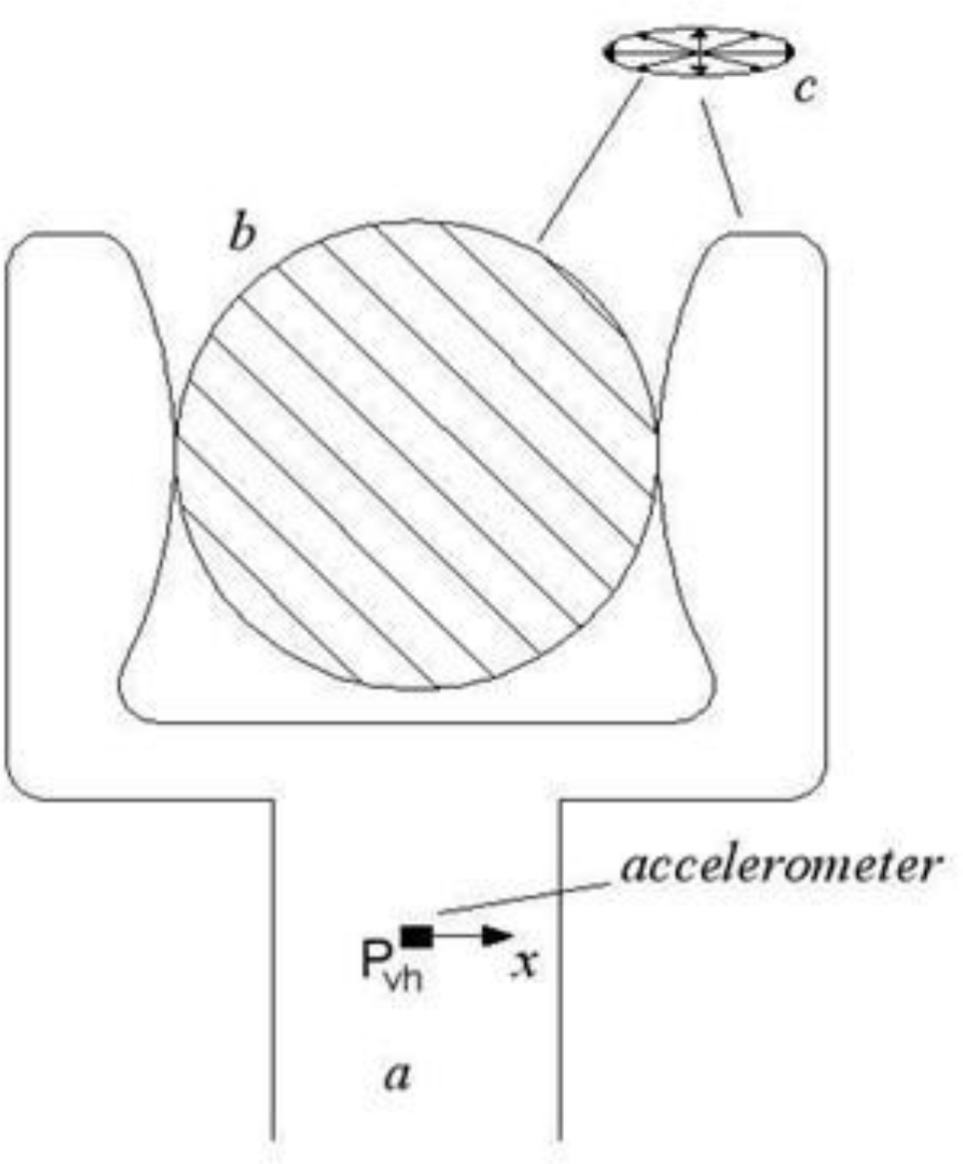
Arrangement of the accelerometers placed on the vibration head **(A)** with *x* axis parallel to the soil; **(B)** cross section of the main branch; **(C)** multidirectional vibrations.

Forced vibratory excitations were produced by the vibrating head of a shaking machine that was wrapped perpendicularly to the primary branches of the selected trees, simulating a typical olive-harvesting operation. One accelerometer was placed on the same P_1_ position as impulse excitation application (see 2.4 section). The gripper of the vibration head was placed approximately 20 cm close to P_1_ (position A in **Figure 1**), in order to better evaluate the amplitude of vibrations transmitted from the machine to the branch. A second accelerometer (P_vh_) was fixed on the vibration head to evaluate the input excitations. The arrangement of this accelerometer data collection for the *x* and *y* axes, which were mutually orthogonal in the plane of the vibration head, with the *x* axis always parallel to the ground (**Figure 3**); only this channel was considered for this accelerometer. The third accelerometer was placed on the same P_3_ position (**Figure 1**) as impulse excitation application (see 2.4 section). It has been employed as a self-propelled shaking machine driven by a 103-kW diesel engine and equipped with a vibrating head located at the end of a 1.8 m telescopic boom which contained both the vibration generator and the gripper. The gripper was wrapped and fastened to the main primary branch that transmitted multidirectional vibrations (**Figure 3**). During the evaluation, the vibration frequency was increased from 11 to 20 Hz within 1 min. Using the aforesaid software, it was found that a spectral analysis on acceleration, expressed as power spectral density (PSD), i.e. the RMS values of the measured accelerations (m s^−2^), at each frequency component in the range 0–250 Hz. PSD indicates the energy (or power) distribution among the frequency components. The PSD values were attained in consideration of the corresponding set of amplitude RMS (m s^−2^) pertinent to the chosen set of not-pruned trees and of the same trees after pruning.

### 2.6 Data analysis

Field-test data collected were analyzed by one and two-way analysis of variance (ANOVA) followed by *post-hoc* testing (SNK protected test) using the R 2.15.0 software (R Foundation for Statistical Computing). The standard error (SE) was also reported.

## 3 Results

### 3.1 Biometry

The re-shaping pruning applied significantly reduced the height and the width of olive trees from averages of 8.5 m to 7.2 m and 10.0 m to 6.6 m, respectively (**Table 1**). The total biomass pruned was considerable (230 kg tree^−1^ as average) and was divided as follows (**Table 2**): 53% with diameter <5 cm (approximately 123 kg tree^−1^) mostly including foliage and shoots, and 47% with >5 cm (average of 107 kg tree^−1^) represented by older branches forming dichotomies over all. Nevertheless, the applied pruning did not affect significantly the shape of the trees (**Figure 1**).

**Table 1:** Tree heights (m) and widths (m) before and after the pruning.

Tree size	Before Pruning	After Pruning
Height (m)	8.5 ± 1.9 a	7.2 ± 0.3 b
Width (m)	10.0 ± 1.0 a	6.6 ± 0.6 b

The standard errors are reported. Letters denote significant differences for each size (SNK test; p= 0.05).

**Table 2:** Fresh weight values (kg tree^−1^) of the total biomass and of that with diameter less/over than 5 cm removed by pruning.

Total biomass (kg tree^−1^)	Biomass with diameter <5 cm (kg tree^−1^)	Biomass with diameter > 5 cm (kg tree^−1^)
230.0 ± 26.4	122.9 ± 34.3	107.0 ± 3.10

The standard errors are reported.

### 3.2 Impulse excitations

**Table 3** reports the RMS values of the amplitude (m s^−2^) applied by the single impulse given to the trunk on the same olive trees before and after the pruning, along three different positions during that time: main branches (accelerometer P_1_), secondary branches (accelerometer P_2_), and tertiary branches (accelerometer P_3_). All accelerometers registered the most elevated amplitudes in the first 30 ms, both before and after pruning; however, the amplitude decays in time were registered with very different modalities.

**Table 3:** Amplitude RMS values (m s^−2^) issued by the single impulse given to the trunk on the same olive trees before and after pruning, along three different positions during the allotted time (ms): main branches (accelerometer P1), secondary branches (accelerometer P2), and tertiary branches (accelerometer P3).

Time (ms)	Before Pruning			After Pruning		
	P_1_	P_2_	P_3_	P_1_	P_2_	P_3_
**30**	0.118a,a	0.019b,b	0.023b,b	0.120a,a	0.110a,a	0.098a,a
**60**	0.010b,a	0.008b,a	0.001b,b	0.089a,a	0.069a,a	0.070a,a
**90**	0.006b,a	0.001b,b	0.001b,b	0.042a,a	0.038a,a	0.029a,a
**120**	0.003b,a	0.001b,b	0.001b,b	0.010a,a	0.009a,a	0.010a,a
**> 150**	0.001b,a	0.001b,a	0.001a,a	0.004a,a	0.003a,a	0.001a,a

The first letter denotes significant differences before and after pruning for each accelerometer position; the second letter denotes significant differences among accelerometer positions at the same pruning status (SNK test; p = 0.05).

On not-pruned trees, in the P_1_ accelerometer, the most elevated amplitudes of 0.118 m s^−2^ reduced very quickly to 0.010 m s^−2^ 60 ms after the impulse. The amplitude slowly decreased to 0.006 m s^−2^ (90 ms), 0.003 m s^−2^ (120 ms), and finally fell to <10^−3^ m s^−2^ after 150 ms. At the level of the P_2_ accelerometers, the amplitude values were significantly lower than those of the P_1_, and the highest amplitude of 0.019 m s^−2^ halved at 60 ms after the impulse; the mean values then fell to <10^−3^ m s^−2^. Finally, the maximum amplitude values registered by P_3_ at 30 ms (0.023 m s^−2^) were similar to those observed on P_2_; however, they collapsed to <10^−3^ m s^−2^ approximately 30 ms after the impulse.

On the same trees, but after the re-shaping pruning, P_1_ accelerometer showed the most elevated amplitudes of 0.120 m s^−2^, similar to those before pruning. In contrast, the amplitudes reduced very slowly and after 90 ms the values remained at 0.010 m s^−2^. After 150 ms, the amplitude fell to 0.004 m s^−2^. The P_2_ amplitude values were similar to the corresponding ones consistently registered by P_1_, with the highest amplitude of 0.110 m s^−2^ at 30 ms and the minimum values 0.003 m s^−2^ 150 ms after the impulse. Moreover, these values were always significantly higher than those registered before pruning. Finally, the P_3_ accelerometer maximum amplitude values were similar to those shown by P_1_ and P_2_ at 30 ms (0.098 m s^−2^), 60 ms (0.070 m s^−2^), 90 ms (0.029 m s^−2^), and 120 ms (0.010 m s^−2^), and they fell under 10^−3^ m s^−2^ 150 ms after the impulse. As for the P_2_ accelerometer, the P_3_ amplitude values were always significantly higher than those registered before pruning, until 120 ms.

To better understand the dynamic responses, the supplementary material (**Figures S1, S2**) reports the decays in the time inside the branches of the vibrations issued by the hits given to the trunk of one representative tree, before and after the re-shaping pruning. The 3-D diagrams interrelate the vibration frequency of the components up to 500 Hz (*x*-coordinate) to the decay in the time up to 270 ms (*y*-coordinate), and the amplitude, reported in logarithmic chromatic scale up to 0.1 m s^−2^ (*z*-coordinate). Graphs are pertinent to the same single impact given to the trees and are found on data registered by the accelerometers P_1_, P_2_, and P_3_.

### 3.3 Vibratory excitations

**Figure 4:**
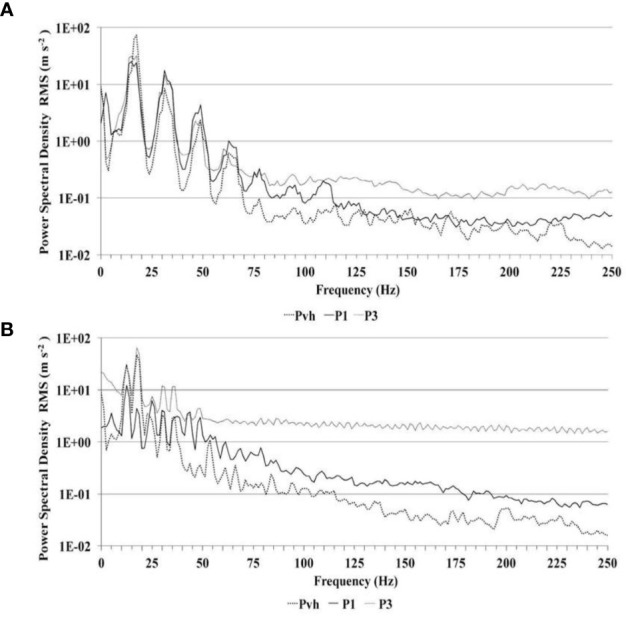
Spectral analysis of accelerations as semi-log graph of the PSD (m s^−2^), obtained with accelerations along the *x*-axis of the accelerometers placed on: i) the vibrating head on primary branch (P_vh_); ii) the closest point to the gripper on primary branch (P_1_); and iii) the furthest point from the gripper on tertiary branch (P_3_), for not-pruned **(A)** and pruned olive trees **(B)**.

**Figure 4** shows the spectral analysis of accelerations as semi-log graphs of the corresponding power spectral density (PSD), expressed as RMS values (m s^−2^), at each frequency component in the range 0–250 Hz, before (**Figure 4A**) and after (**Figure 4B**) pruning on the same tree. PSD values were obtained from accelerometers placed on the vibrating head (P_vh_), closest to the gripper (P_1_), and furthest from the gripper (P_3_). In general, the PSD values were high at low frequencies and gradually attenuated as the frequency components increased. The PSD at the vibration head (P_vh_ accelerometer) was mainly allocated on the low frequencies, corresponding to the stimulation frequencies, while the highest value was assigned to the frequency around 17.5 Hz in both cases (**Figures 4A, B**). Beyond these frequencies the PSD values of the P_vh_ accelerometer decreased differently before and after pruning. In particular, for unpruned trees, they ranged from 0.1 m s^−2^ to 1 m s^−2^ between 50 and 70 Hz. In contrast, after pruning, the PSD values were found in the same range during a larger interval of frequencies, between 40 Hz and 115 Hz (**Figures 4A, B**). The acceleration values pertinent to P_1_ and P_3_ accelerometers attenuated as the frequency increased. The relative curves of spectral distribution generally developed above the curve, relative to the P_vh_ accelerometer, and they were significantly different before and after pruning (**Figures 4A, B**). Before pruning, the PSD graph pertinent to accelerometer P_1_ displayed a sinusoidal trend for frequencies up to approximately 70 Hz, and the values were similar to those of the vibration head (P_vh_) for frequencies higher than approximately 115 Hz, when the values become less than 0.1 m s^−2^ (**Figures 4A**). After pruning, the PSD graph displayed a sinusoidal trend for frequencies up to approximately 40 Hz, when the values ranged from 1 m s^−2^ to 10 m s^−2^; however, for frequencies higher than approximately 190 Hz, the values were less than 0.1 m s^−2^ (**Figure 4B**). Finally, before pruning, the PSD values of the accelerometer P_3_ showed a sinusoidal trend for frequencies up to approximately 70 Hz, similar to the values of P_vh_ and of P_1_, and the values ranged from 0.1 m s^−2^ to 1 m s^−2^ (**Figure 4A**). Over 70 Hz, the PSD values remained close to 0.1 m s^−2^ until 250 Hz. After pruning, the curve developed over the curve of the P_vh_ and P_1_ accelerometers and the PSD values were consistently ranging from 1 m s^−2^ to 10 m s^−2^ at all frequencies (**Figure 4B**).

## 4 Discussion

This study reports the first data about the dynamic behavior of centuries-old tree skeletons, in the context of very large-sized olive trees, while taking into account the effects of two different vibrations application modes: a realistic one represented by the system vibration head-tree, originated when the gripper of a shaking machine wraps and fastens the main branch of an olive tree, and a more speculative one, represented by a single impulse of a short-duration force originated by a hammer. An impulse excitation is an example of non-periodic force and any common occurrence that causes such an excitation is an impact; thus, exciting a broad range of frequencies (Du et al., 2012; James et al., 2014). The results revealed that the impulse excitations produced free vibrations inside the branches, which damped faster inside the not-pruned trees than in the same trees undergone re-shaping pruning. The trees that underwent re-shaping pruning conveyed frequencies higher than the same trees not yet subjected to re-shaping pruning; moreover, decay times were significantly lower and amplitude values were one order higher, particularly in the tertiary branches. In general, the trees had a very similar dynamic response to applied vibratory excitations among them, both before and after the re-shaping pruning. The PSD at the vibration head was largely allocated on the low frequencies, probably because at this value, corresponding to 1050 rev/min of the eccentric masses, the vibration force reached the greatest amplitude (**Figures 4A, B**). The centuries-old olive trees subjected to re-shaping pruning highlighted greater stiffness, demonstrated by higher decay times (120–150 ms) and higher vibration amplitudes (>10^−1^ m s^−2^), at the same frequency under impulse excitation and by power spectral density one logarithmic order higher under vibration excitation in the tertiary branches. Similar spectral analysis of accelerations of vibrations were reported by Caprara and Pezzi (2011) on grapes, where in the sinusoidal trend in the frequencies ranged from 0–50 Hz and the curves increased as distance from the vibrations application increased (**Figure 4**). However, the dynamic behavior has resulted in significantly different values before and after pruning. The main effect of the re-shaping pruning was a 10-fold increase in PSD in P_3_ of pruned trees (range 1.0–10 m s^−2^) compared to that of to not-pruned ones (range 0.1–1.0 m s^−2^) at a frequency >50 Hz. This was due to a strong increase in the rigidity of the trees, which allowed better transmission of vibrations inside the branches. These results agree with other studies also conducted on different tree species (pistachio, almonds, and coffee) even if on not centennial trees (James, 2003; Láng, 2006; Polat et al., 2007; Du et al., 2012; He et al., 2013; Gonçalves et al., 2016).

Tests performed on very large-sized centuries-old olive trees showed significant improvement in vibration transmissions on pruned with respect to not-pruned trees. However, the re-shaping pruning applied was not invasive, involving only the tertiary branches. Either impulse and vibratory excitations tests clearly highlighted the best dynamic response of the trees after the re-shaping pruning, both in terms of vibration damping and amplitude/power spectral density transmitted. The free vibrations inside the branches caused by impulse excitations damped quickly inside the not-pruned trees in comparison with the same trees that underwent re-shaping pruning. Conversely, the vibrations created by the shaking machine were significantly damped inside the not-pruned trees, probably due to the considerable mass of the branches and the remarkable aerodynamic drag forces of the foliage in the air (Tsatsarelis, 1987; Inman, 1994). Regardless of vibratory excitation modalities, the transmission of vibration among the branches of very large-sized olive trees was affected by damping effects linked to different agents (leaf drags, internal energy losses within the wood and root/soil system, and so on), which produce dynamic responses that may not be predictable. The re-shaping pruning removed foliage and branches in nearly equal parts (**Table 1**; **Figure 1**); moreover, it acted primarily by reducing tree width (−34%) rather than tree high (15%) (**Table 1**) and by thinning hanging branches, dichotomies, and foliage (**Table 2**). The considerable pruning biomass values measured are consistent with Velázquez-Martí et al. (2011) findings in Spain under similar agronomical conditions (tree size, tree age, tree density, and water availability), when heavy pruning is performed. The significant damping of vibrations of P_1_ and P_3_ branches in unpruned trees was primarily due to the remarkable aerodynamic effect of the foliage (Tsatsarelis, 1987; Inman, 1994). In contrast, the branches of the pruned trees had higher stiffness and dynamic response under vibratory excitations concerning P_1_ and P_3_ positions, which highlighted a greater reaction to the entrance of vibration transmitted by the gripper. Thus, the greater stiffness of the pruned trees, the higher amplitude suitable for producing disconnection force between pedicel-fruit system (Láng, 2006). Moreover, these high amplitudes should be suitable to achieve the maximum fruit-removal efficiency (Hoshyarmanesh et al., 2017; Castillo-Ruiz et al., 2018). The mechanical detachment of olives from trees is a complicated occurrence and only certain combinations of amplitude and frequency of forced vibrations are applicable (Spatz et al., 2007; Vieri and Sarri, 2010). This complexity is stressed if the vibration is applied to areas remote from the fruits, as the vibration has to be transmitted to the fruit through the skeleton of the tree (Du et al., 2012; Zhou et al., 2013). As the PSD values indicated the energy (or power) distributed among the different frequency components along the branches, these data support the hypothesis that a more rational pruning could significantly increase the vibration transmission, with useful implications on efficiency of branch-shakers machines.

Our results showed that the transmission of vibrations is quite complex in the case of centuries-old olive trees; this may increase the knowledge about their dynamic behavior. A more rational pruning applied to ancient large-sized olive trees significantly increased the vibration transmission under both impulse and vibratory excitation forces, without affected their typical “look”. Moreover, these insights are helpful in turn in achieving maximum fruit-removal efficiency. Further studies are ongoing to assess the effects of pruning on structural stiffness of ancient trees, for other olive cultivars and in different growing conditions, in order to increase the vibrations transmission efficiency of branch-shaker harvesting machines. The sustainability of this dominant key portion of the Mediterranean landscape and cultural heritage is at stake: it would like to keep and sustain this heritage, by increasing oil quality and olive growers profitability.

## Data availability statement

The raw data supporting the conclusions of this article will be made available by the authors, without undue reservation.

## Author contributions

Conceptualization and methodology by FV, GV, SC, and SP. Formal analysis, investigation, and data curation by FV, SC, and SP. Writing (original draft preparation) by FV, SC, and SP. Writing (review and editing) by FV, GV, SC, and SP. Supervised by SC and SP. All authors contributed to the article and approved the submitted version.

## Footnotes

1 IOC. International Olive Council. http://www.internationaloliveoil.org/estaticos/view/136-countryprofiles
