# Supplementary material for: Re-shaping pruning improves the dynamic response of centuries-old olive trees to branch-shaker vibrations application
Source: Front Plant Sci. 2023 Apr 21;14:1155120. doi: 10.3389/fpls.2023.1155120 (PMC10162015; doi:10.3389/fpls.2023.1155120)
Supplement: Supplementary file 1 [file DataSheet_1.docx]

Supplementary Material

Re-shaping pruning improves the dynamic response of centuries-old olive trees to branch-shaker vibration application

**Salvatore Camposeo*, Francesco Vicino, Gaetano Alessandro Vivaldi, Simone Pascuzzi***

Department of Soil, Plant and Food Science, University of Bari Aldo Moro, Bari, Italy

*** Correspondence:**Simone Pascuzzi
[simone.pascuzzi@uniba.it](mailto:simone.pascuzzi@uniba.it)

Salvatore Camposeo

s[alvatore.camposeo@uniba.it](mailto:alvatore.camposeo@uniba.it)

## Supplementary Figures


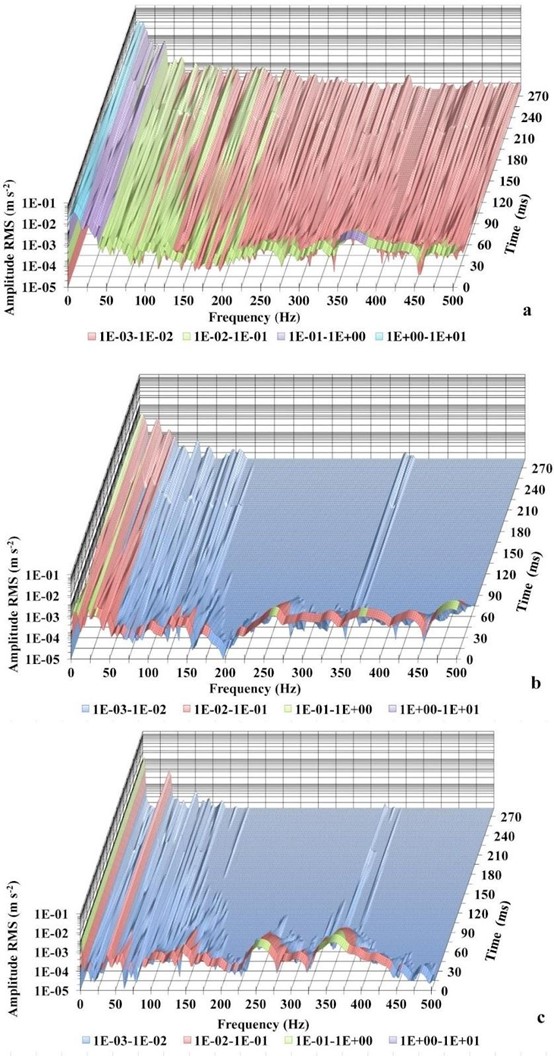


**Figure S1.** Variations of frequency (Hz) and amplitude rms (m s^-2^) in the time (ms) of the vibrations issued by the single impulse given to the trunk along the primary branch (a), secondary branch (-b) and tertiary branch (c) - P_1_, P_2_ and P_3_ accelerometers, respectively. Data are referred to a representative olive tree before pruning.


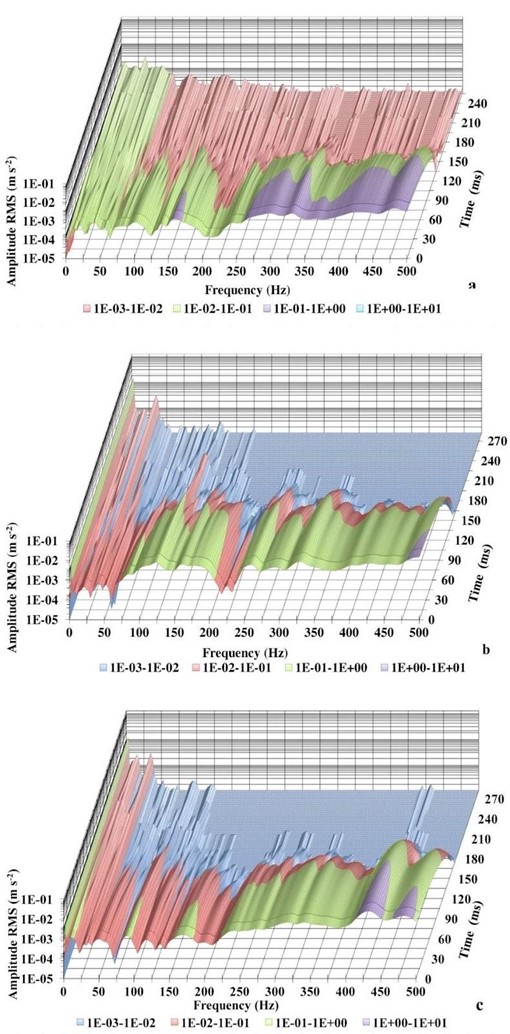


**Figure S2.** Variations of frequency (Hz) and amplitude rms (m s^-2^) in the time (ms) of the vibrations issued by the single impulse given to the trunk along the primary branch (a), secondary branch (-b) and tertiary branch (c) - P_1_, P_2_ and P_3_ accelerometers, respectively. Data are referred to a representative olive tree after pruning.
